# Seizures in Children with Influenza during the 2022–2023 Winter Season, a Case Series

**DOI:** 10.3390/clinpract14010014

**Published:** 2024-01-19

**Authors:** Francesca Peranzoni, Carine Martins, Sébastien Lebon, Pierre Alex Crisinel, Marie-Helena Perez

**Affiliations:** 1 Pediatric Intensive and Intermediate Care Units, Service of Pediatrics, Women-Mother-Child Department, Lausanne University Hospital, 1005 Lausanne, Switzerland; carine.ferreira-martins@chuv.ch (C.M.); marie-helene.perez@chuv.ch (M.-H.P.); 2 Unit of Pediatric Neurology and Neurorehabilitation, Service of Pediatrics, Women-Mother-Child Department, Lausanne University Hospital, 1005 Lausanne, Switzerland; 3 Unit of Pediatric Infectious Diseases and Vaccinology, Service of Pediatrics, Women-Mother-Child Department, Lausanne University Hospital, 1005 Lausanne, Switzerland; pierre-alex.crisinel@chuv.ch

**Keywords:** neurotropism, influenza, pediatric, seizures

## Abstract

Influenza is a viral infection presenting with general symptoms such as fever, headache, fatigue, and involvement of airways or the gastrointestinal tract. The nervous system may be involved, but less frequently. These neurological complications remain challenging to diagnose; moreover, no guidelines for management and treatment exist. Therefore, when presenting with neurological symptoms, patients undergo invasive diagnostic procedures and empirical treatments before making the correct diagnosis. During the winter of 2022–2023, four children between nine months and nine years of age were admitted to the Lausanne University Hospital, Switzerland, complaining of influenza and neurological complications. This report presents the symptoms of neurological manifestation and the treatment management of the four patients. All the legally authorized representatives gave their written informed consent before study inclusion.

## 1. Introduction

Influenza, a negative-sense single-stranded RNA virus, belongs to the *Orthomyxoviridae* family and is classified into three antigenic types: A, B, and C. Influenza A and B viruses possess eight RNA segments that encode 10 and 11 proteins, respectively. These viruses are responsible for influenza epidemics and pandemics. Influenza C, on the other hand, typically causes milder and less frequent infections [1].

Influenza commonly leads to respiratory diseases ranging from mild to severe in nature [2]. Diagnosis is typically based on samples collected from the nasopharynx, throat, or saliva [3]. Central nervous system (CNS) involvement in children has been primarily associated with influenza A [4,5].

Febrile seizures are a frequent complication, followed by influenza-associated acute encephalopathy (IAAE), stroke, and meningitis [6]. Additional neurological complications comprise post-influenza encephalopathy, Reye’s syndrome, Klein–Levin syndrome, post-encephalitic Parkinson’s disease, encephalitis lethargica [7], and Guillain–Barre syndrome [8]. Those complications are four times more frequent in patients with chronic neurological or neurodevelopmental disorders such as intellectual disability, cerebral palsy, or epilepsy [9]. The exact mechanism behind the emergence of neurological complications in influenza infections is still not understood. However, it has been observed that certain patients with particular genetic predispositions produce proinflammatory cytokines that lead to vascular endothelial damage and heightened permeability of the blood–brain barrier during influenza infections [10,11]. Two studies have indicated that most patients with clinical and neuroradiological evidence of CNS involvement exhibit low CSF pleocytosis [6,12]. This suggests CNS injury may be associated with inflammatory or immune-mediated responses [13]. Recently, Influenza A was determined to have been playing a role in disrupting the oligodendrocytes homeostasis in mice models [14]. Neuroradiologic imaging techniques, including computed tomography (CT) and magnetic resonance imaging (MRI), along with neurological clinical examination, aid in assessing the severity of the disease.

This report examines the presentation and management of influenza-related neurological complications in four children admitted to the intermediate care unit of the Lausanne University Hospital during the winter season of 2022–2023.

## 2. Case Series

### 2.1. Case 1

A 23-month-old girl was admitted after experiencing three instances of (complex) febrile seizures in 24 h. During the initial seizure, she had a focal onset with a right-side head and mouth deviation, loss of contact, followed by bilateral propagation, leading to a generalized clonic seizure. The second and third seizures were characterized by generalized clonic activity. Each seizure lasted for less than a minute and was accompanied by nasal congestion and cough that had been present for 48 h. The girl’s condition returned to normal after the seizures. Brain injury was not reported in medical history.

Table 1 presents her vital parameters (VPs) and laboratory workup (LW). The polymerase chain reactions (PCRs) included in the hospital standard panel (Table 1) for cerebrospinal fluid (CSF) were negative. A potential CNS infection was suspected, leading to the initiation of intravenous (IV) ceftriaxone at a dosage of 100 mg/kg/day and acyclovir at a dosage of 16 mg/kg/day. The nasopharyngeal swab confirmed the presence of Influenza B. To minimize the duration and severity of the infection and the likelihood of adverse neurological effects, a five-day course of Oseltamivir at a dosage of 30 mg twice daily was administered. Ceftriaxone and acyclovir were discontinued after 48 h due to the negative CSF culture results. Influenza was the only pathogen detected, and it was considered responsible for the neurological symptoms.

Due to favorable clinical evolution with non-recurrence of neurological manifestations, the patient was discharged after 48 h and was scheduled for a follow-up at the pediatric neurology outpatient clinic. Neurological examination and her electroencephalogram (EEG) one month after the hospital discharge were normal, and no further examinations were scheduled.

### 2.2. Case 2

A 15-month-old boy was transferred from a peripheral hospital following repeated (complex) febrile seizures within the past 24 h. Ocular revulsion, clonic generalized movements, and axial hypotonia characterized these seizures. The episodes occurred alongside nasal congestion and bilateral otitis media (OM). The boy’s neurological status remained normal during and after the seizures (please refer to Table 1 for the listed VPs and LW). A nasopharyngeal smear confirmed an infection of Influenza A. A brain CT scan revealed no abnormality. Although three attempts were made, the lumbar puncture remained unsuccessful. However, based on the clinical findings (excellent neurological and general clinical status) and imaging results, neurological infection was ruled out, and no other attempts were made to obtain an LP. Influenza was considered responsible for the symptoms.

The boy received a five-day course of oseltamivir. OM was treated with a ten-day course of amoxicillin as the physical examination suggested a bacterial etiology. He was discharged on the second day after examination, as his physical condition was good.

One month later, during an airway infection, the boy experienced three non-febrile seizures. A respiratory panel yielded negative results, with low CRP levels (8.3 mG/L). He was given a prophylactic dose of levetiracetam at 10 mg/kg twice daily for one day. His condition immediately improved, and his neurological status returned to normal. Two days later, he was discharged with emergency intranasal midazolam treatment, which could be administered at home by caregivers. A follow-up was arranged in pediatric neurology. The EEG results were normal, and no further follow-up or treatment was deemed necessary.

### 2.3. Case 3

A 9-year-old boy was admitted following a generalized motor seizure lasting less than three minutes. The seizure was characterized by hypertonia, clonic movements in all four limbs, and trismus. He had been experiencing a fever of around 39.5 °C for the past 24 h. At the age of three, he had a history of one febrile seizure; however, there was no family history of epilepsy. During the clinical examination, abnormal pulmonary auscultation and pharyngitis were observed. Please refer to Table 1 for the reported workup. The boy’s neurological status was normal upon admission, with no signs of meningeal irritation.

He received IV ceftriaxone at a dosage of 100 mg/kg/24 h and acyclovir at a dosage of 16 mg/kg/d for 48 h. The BCT scan was normal. CSF PCRs and cultures, as well as blood cultures, were all sterile. The respiratory viral panel indicates the presence of influenza A, and a positive streptococcal was also observed. Influenza was retained as the responsible pathogen of seizures after the exclusion of other possible causes, such as brain injury, brain tumor, bacterial or viral meningitis, and encephalitis. To manage the infection, oseltamivir was administered at a dose of 30 mg/twice daily for five days, like in case 1.

The boy was discharged after 72 h with a scheduled follow-up and an EEG at one month. The EEG results showed no anomalies; hence, no other follow-up was deemed necessary.

### 2.4. Case 4

A 9-month-old boy presented with a complex febrile seizure characterized by a lack of contact, a fixed gaze, hypertonia of all limbs, followed by prolonged hypotonia. The seizure lasted 15–30 min and occurred in the context of an ear–nose–throat infection, with left OM and a fever of approximately 39 °C, persisting for one week. The seizure ceased after three doses of intranasal midazolam at a dosage of 0.2 mg/kg/dose.

To rule out any complications of the OM leading to CNS infection, a brain CT scan was performed, which yielded normal results excluding, among other things, brain traumatic injury and cerebral compressive syndrome. A laboratory workup was conducted as shown in Table 1. Influenza B was detected through the nasopharyngeal swab.

Empirical treatment for bacterial or herpes meningitis was initiated with IV ceftriaxone at a dosage of 100 mg/kg/24 h and acyclovir at a dosage of 16 mg/kg/day. The CSF PCRs and cultures yielded sterile results, leading to the discontinuation of anti-infectious treatments after 48 h. A ten-day course of amoxicillin was prescribed to address the otitis with suspected bacterial etiology. Influenza was considered responsible for the neurological symptoms in this patient.

The patient was discharged on the third day, and a follow-up appointment was scheduled with the family doctor.

## 3. Discussion

During the winter of 2022–2023, the IMCU of the Lausanne University Hospital, Switzerland, admitted four children with influenza-associated seizures.

Three patients displayed signs of airway infection, while two had otitis media. All four children experienced high fever. Complex seizures were the only neurological symptoms observed. None of the patients had a familial history of epilepsy or neurological problems, and none had been vaccinated against influenza.

Complex febrile seizures and fever showed a potential severe CNS infection. Therefore; routine investigations were carried out, including blood tests, LP, nasopharyngeal swabs, and complementary imaging. Brain CT was the chosen imaging modality, as MRI was not necessary considering the rapid favorable progression of the patients, longer execution time, and the need for sedation in young patients. Empiric treatments for suspected bacterial or herpetic CNS infection (ceftriaxone at a dosage of 100 mg/kg/24 h and acyclovir at a dosage of 16 mg/kg/day) were administered in three cases.

After undergoing all the above-mentioned examinations, two patients were found to be infected with Influenza A, while the other two had Influenza B. The results of viral PCR from the nasopharyngeal swab took 6 to 48 h to be received. Contemporary complementary investigations excluded other differential diagnoses such as brain injury, tumor, bacterial and viral meningitis, or encephalitis.

One child could not undergo LP and did not receive the empiric treatment due to his excellent clinical evolution, normal imaging investigations, and a positive influenza swab. This case should be considered as an exception to the routine practice. No guidelines suggest that early detection of the influenza virus in a child presenting with complex febrile seizures and fever permits the clinician to exclude other CNS infections.

Influenza was considered responsible for neurological symptoms in all the patients.

Three children who had experienced symptoms for less than two days were treated with oseltamivir at a dosage of 30 mg twice daily for five days. The effects of this treatment on the outcome were uncertain. Oseltamivir had shown efficacy when administered within 48 h of the symptom onset, reducing the duration of symptoms such as fever, cough, and otitis media [15]. While no study has examined the impact of antiviral treatment on influenza-associated CNS manifestations, reports have indicated the use of antivirals in certain neurologic complications of influenza [12,16]. Therefore, proposing oseltamivir to patients presenting with symptoms within 48 h and concomitant neurological manifestations was deemed reasonable. Home discharge was considered after the patients presented a favorable evolution and according to the caregivers’ understanding of the situation. Follow-up visits were planned for all the patients, either with pediatric neurologists or their pediatrician, to conduct additional examinations such as EEG when necessary or for a clinical evaluation. All four children had normal clinical status, and those who underwent EEG had normal results. One patient had been hospitalized twice for seizures, suggesting a low epileptogenic threshold.

Personal predisposition to develop febrile seizures can be related to mutations in chromosome sections, especially when febrile seizures run in families [17]. Another hypothesis sustains that the predisposition to febrile seizures is associated with low glutamine synthetase (GS): a glial enzyme with a fundamental activity in the neuronal excitation neurotransmission pathway [18]. In the future, further studies will be required to test this hypothesis in detail.

Nowadays, there is a lack of guidelines regarding the prevention, management, and treatment of febrile seizures and encephalitis. Only small case series exist to provide clinicians with suggestions on handling influenza-related neurological complications [12,16]. Moreover, the pathogenic pathway for influenza neurological involvement is not yet understood, even if recent studies have determined that the Influenza A virus disrupts the oligodendrocytes’ homeostasis in mice models [14]. Further studies are needed to understand if influenza viruses can lead to an imbalance in glial cell excitatory and inhibitory functions with a similar pathway to epilepsy [19]. On the other hand, the mechanism through which febrile seizures are provoked during an infection is well known. The inflammatory response during an infection produces cytokines that activate the microglia. Activated microglia cells secrete IL1β, which induces fever by interacting with hypothalamic neurons. Abnormally increased IL-1β levels also progressively increase excitatory (glutamatergic) neurotransmission and decrease inhibitory (GABAergic) neurotransmission, thus mediating the pathogenesis of convulsions [20,21]

While febrile seizures are common during viral infection in childhood, it is essential to detect those cases related to well-known neurotropic viruses, such as influenza, which can potentially lead to life-threatening complications.

This case series is valuable because it presents an approach to managing children with seizures and influenza. It highlights the importance of rapid diagnostic testing for influenza infection in seizure management, particularly during influenza season. It offers clinicians a low level of evidence-based medicine for all-day practice.

However, due to the small cohort, it is impossible to determine whether antiviral treatment influenced the outcome or if a positive influenza swab in a child presenting with seizures and no other clinical complaints could prevent the need for LP and empiric therapies.

## 4. Conclusions

Additional research is required to gain a deeper understanding of the pathogenesis of these conditions and to develop more effective diagnostic methods and protocol strategies for better managing influenza-related neurological complications. This will help in avoiding unnecessary invasive tests and empiric treatment.

## Figures and Tables

**Table 1:** BPM, beat per minutes; RR, respiratory rate; T, temperature; BP, blood pressure; Na+, sodium (normal range between 135 and 145 mmol/L); K+, potassium (normal range between 3.4 and 5.2 mmol/L); Ca++, calcium (normal range between 2.20 and 2.60 mmol/L); Ph, phosphorus (normal range between 1.12 and 1.45 mmol/L); Mg++, magnesium (normal range between 0.75 and 0.95 mmol/L); CRP, C reactive protein (normal range <3 mG/L); Lecocytes in blood normal range between 4 and 10 G/L; Hemoglobin normal male range between 117 and 157 G/L, normal female range between 133 and 177 G/L; Neutrophils in blood percentage normal between 50% and 70%; Proteins in cerebrospinal fluid normal range between 150 and 690 G/L; Glucose CSF/blood glucose, normal range between 0.41 and 0.88; Leucocytes in CSF normal range between 0 and 5 cells (all mononuclear); Erythrocytes in CSF normal range 0.

	Case 1	Case 2	Case 3	Case 4
Vital signs	138 bpm, RR 28/min, BP 62/58 mmHg, T 36.7 °C at admission	119 bpm, RR 28/min, BP 107/52 mmHg, T 36.7 °C at admission	134 bpm, RR20/min, BP 109/66 mmHg, T 39.5 °C at admission	200 bpm, RR 40/min, BP 100/60 mmHg, T 39 °C at admission
Blood test	Na+: 139 mmol/L Ca++, Mg++: Normal Leucocytes 10.7 G/L, Hemoglobin 119 G/L CRP 39 mG/L	Lc 23.1 G/L Leucocytes 14.8 G/L Hemoglobin 114 G/L CRP 39 mG/L	Na+: 134 mmol/L, K+: hemolysis CRP 14 mG/L Leucocytes 7.3 G/L	Na+: 136 mmol/L, K+: 4.4 mmol/L, Ca++ 2.45 mmol/L, Ph 1.73 mmol/L Leucocytes 14.2 G/L Neutrophils 79% CRP 14 mG/L
Lumbar puncture (CSF) and Polymerase chain reactions (PCRs) (Neisseria meningitidis, Listeria monocytogenes, Streptococcus pneumoniae, Haemophilus influenza, Escherichia coli K1, Streptococcus agalactiae, Enterovirus, Herpes simplex 1 and 2, Varicella-zoster, Cytomegalovirus, HHV6, Parechovirus)	Proteins 201 mG/L Glucose CSF/blood glucose: 3.9 mmol/L/5.2 mmol/L = 0.75 Leucocytes 3 × 10^6^ /L (75 lymphocytes, 25 Monocytes/macrophage) Erythrocytes 70 × 10^6^ /L PCRs negatives	Not done	Proteins 212 mG/L Glucose CSF/blood: 4 mmol/L/5.6 mmol/L = 0.71 Leucocytes < 1 × 10^6^ Erythrocytes 0 PCRs negatives	Proteins 220 mG/L Glucose CSF/blood: 4.7 mmol/L/5.7 mmol/L = 0.82 Leucocytes 0 Erythrocytes 0 PCRs negatives

## Data Availability

Data are not available due to privacy restrictions.
